# The Global Challenge of Child Injury Prevention

**DOI:** 10.3390/ijerph15091921

**Published:** 2018-09-04

**Authors:** David A. Sleet

**Affiliations:** 1 TJFACT Consulting, Atlanta, GA 30303, USA; davidasleet@gmail.com; 2 Rollins School of Public Health, Emory University, Atlanta, GA 30322, USA

*“If a disease were killing our children at the rate unintentional injuries are, the public would be outraged and demand that this killer be stopped”*

$\begin{array}{c} \text{-}C.\;\text{Everett Koop, MD}\\ \text{13th Surgeon General of the United States of America (1982 to 1989).} \end{array}$

## 1. Background 

The health of children has changed significantly during the past 50 years. Widespread immunization programs have nearly eliminated the threat of infectious diseases, such as polio, diphtheria, and measles. However, a major public health problem that continues to threaten the health of all children has no vaccine: injury. Child injury represents one of the most immediate public health threats, resulting in the death of nearly 2000 children under age 14 every day around the world. (http://www.who.int/healthinfo/global_burden_disease/estimates/en/). According to Margaret Chan, former Director General of the World Health Organization (WHO), and Ann Veneman, former Executive Director of the United Nations Children’s Fund [1]:

Once children reach the age of five years, unintentional injuries are the biggest threat to their survival. Unintentional injuries are also a major cause of disabilities, which can have a long-lasting impact on all facets of children’s lives: relationships, learning and play. Among those children who live in poverty, the burden of injury is highest. Child injuries have been neglected for many years and are largely absent from child survival initiatives presently on the global agenda. The World Health Organization, the United Nations Children’s Fund and many partners have set out to elevate child injury to a priority for the global public health and development communities.

(p. VII)

In the compilation of 23 papers from this Special Issue of the *International Journal of Environmental Research and Public Health (IJERPH) * [2], we review the burden of childhood injuries, discuss effective interventions applied to specialized target groups around the world, and focus on methods to bring interventions into practice settings with an eye toward the use of theoretical approaches and program evaluation. Some papers in this Special Issue focus on epidemiology and surveillance, some on clinical and community intervention and many generate new knowledge that may help curb the problem in the future. Some papers discuss the need for translational research from discovery to delivery, and in transmitting knowledge for change from one setting to another. All except two focus exclusively on unintentional injuries.

## 2. Introduction

Child injury is not new to medical science and public health, but the professions have been slow to recognize opportunities for prevention. Historically, examples of treating cases of head and foot injuries can be found on papyrus dating back to 1600 BC, and Hippocrates studied wounds and fractures, and their causes, as far back as 500 BC [3]. Modest public health and environmental efforts addressing child injuries began in the 1940s and 1950s, but it was not until the 1960s that concentrated efforts in the U.S. and elsewhere were made to collect and use data, formulate policies, and implement best practices to reduce childhood injuries [4,5].

Injury epidemiology and prevention research have recognized the growing burden of childhood injury, but it has taken them a long time to address it. Glied [6] provided data from Medline about the increase in the production of research articles in the United States. Glied found that the annual number of publications grew from an average of 19.1 during the period of 1966–1975, to 58.4 during the period 1993–1997. Publications on childhood injury steadily grew in both the number of articles published per year and as a proportion of all publications on children’s issues during that 31-year period. 

Since that time, publications of research articles, books, and commentaries on child injury and prevention have mushroomed. According to a recent analysis of annual publication in infant, child and adolescent child injury conducted by Lawrence [7] using the SafetyLit database of citations not listed in Medline [8], he uncovered a total of 55,467 publications from 1966–2017, and 26,648 publications from 1966–1997 compared with Glied’s 187 publications during the same period (1966–1997). Despite what might be a gross underestimate of annual publications by Glied (the inclusion and exclusion criteria may have been different), it is clear that the topic of child and adolescent injuries has become increasingly popular during the past 50 years.

The current compilation complements two recent special issues of *IJERPH* on child injury [9,10] that focus on the ways child injury has emerged as a public health concern, and a recent special issue on child injury in the *Journal of Pediatric Psychology* [11] focusing on child psychology and injury. These Special Issues along with the current one, further illustrate the growth and interest in addressing the prevention of child injury.

## 3. Injury and Human Development 

Great progress has been made in increasing child survival in the 20th Century. From 1915 through 1997 in the United States, the infant mortality rate declined greater than 90% to 7.2 per 1000 live births [12,13]. Global child mortality fell from 18.2% in 1960 to 4.3% in 2015 [14]—an unprecedented achievement in child health. These reductions were largely the result of environmental interventions, improvements in nutrition, advances in clinical medicine, improvements in access to health care, improvements in surveillance and monitoring of disease, increases in education levels, and improvements in standards of living [13,15].

Glied [6] claims, however, that a majority of the reductions in infant and child mortality in the U.S. are the result of reductions in injury:

A growing share of the reduction in child mortality, however, stems neither from medical advances nor from immunization campaigns. Rather, the most important contributor to reductions in mortality since 1970 has been a sharp decline in the rate of child mortality from unintentional injury or accidents. Among children under five, that rate dropped from 44 deaths per 100,000 population in 1960 to 18.6 deaths per 100,000 population in 1990. Among children ages five through nine, the rates dropped from 19.6 to 9.8.

(p. 511)

It is unclear, however, what proportion of overall reduction in child mortality is due to reductions in injury deaths compared to reductions in deaths from all other causes. But as Meddings [16] points out, while protecting the health of infants in the first year of life should remain a major public health priority in many parts of the world the majority of children who survive their first year of life are still confronted by the threat of injuries; “As children move from infancy to older ages their threats to health do not just recede; they undergo a qualitative shift which includes an increasing preponderance of injury threats. The current palette of child survival programming has not evolved in any substantive way to address these” (p. 69)

The prevention of injuries to children should be part of every child survival program, every maternal child health program, and incorporated into every primary health-care system—in both high income and low- and middle-income countries. In addition to preventing injuries, programs need to improve emergency services and acute care for the injured child and strengthen rehabilitation programs.

## 4. Injury Intent and Mechanisms

Childhood injuries can be classified by intent: unintentional or intentional, and by mechanism or cause. Traffic injuries, burns, sports-related injuries, falls, drowning, suffocation, and poisonings are common causes of unintentional injuries. Injuries due to assault, self-inflicted violence, such as suicide, and war are classified as intentional injuries. Most injuries to children by these mechanisms are unintentional. While the collection of papers in this volume focuses mainly on unintentional injuries in childhood, two papers discuss violence: one on child abuse in the slums of Kampala in Uganda [17] and another on the epidemiology of child unintentional injury and violence in the USA [18]. Papers in this compilation include injury mechanisms and outcomes such as motorcycle-related head injuries, drowning, motor vehicle crashes, physical abuse, burns, sports injury, traumatic brain injury (TBI), and musculoskeletal injuries.

## 5. Child Injury Burden

In 2016, WHO estimated that over 644,855 children under the age of 15 were killed by an injury and between 10 million and 30 million more suffered a non-fatal injury [19]. Moreover, 95% of injury deaths occurred in low- and middle-income countries. According to Dr. Sabastian Van As, who heads the trauma unit at the Red Cross War Memorial Children’s Hospital in Cape Town, South Africa, “The world is a dangerous place for children … even more so in poor countries. A child growing up in Cape Town, South Africa is 25 times more likely to be admitted to hospital for an injury than a child in Birmingham, UK” [20].

In the European region, where injuries are the leading cause of death in children aged 5–19, nearly 42,000 children and adolescents aged 0–19 years die annually from unintentional injuries; five out of six of these deaths occur in low- and middle-income countries. Irrespective of income, the burden falls disproportionately on children from the most disadvantaged groups and in the most impoverished environments [21].

In the United States, about 19 children ages 1–19 die every day from an unintentional injury. One in four children annually will be injured severely enough to miss school or work or require medical attention or bed rest. In 2016, for every unintentional injury death to those ages 0–19 there were approximately 33 hospitalizations and 1053 emergency department visits [22].

A large proportion of these unintentional injuries (for example, burns, suffocation, poisoning, and falls) occurred in or around the home while others occurred in the community (for example transportation-related injuries, drowning and sports injures). These injuries represent a serious burden to the injured person and their family and represent a tremendous economic and community burden; yet, most are predictable and preventable.

Globally, injuries to children are also a large and growing problem. According to the 2013 Global Burden of Disease Study [23] road traffic injuries were the leading cause of death among adolescents around the world. Overall road traffic deaths have actually increased globally by about 13% since 2000 [24]. In response to this crisis, the United Nations Sustainable Development Goals (Goal 3.6) have set a target for reducing traffic injuries and deaths by 50% by 2020 [25]. However, with increasing motorization in many countries, traffic deaths are likely to worsen before they improve [26].

Worldwide, drowning is among the 10 leading causes of death among children and adolescents with children under age 5 disproportionately at risk [27]. Key risk factors in low- and middle-income countries include access to water bodies, such as ponds, ditches, rivers, lakes, and dams. In high-income countries drownings often occur in swimming pools that are not fenced and where there is access to unsupervised coastal waters and beaches [27,28,29]. Older children and adolescents usually drown during non-recreational or daily activities in developing countries but during recreational activities in developed countries [28]. Risk of death from drowning is especially high in rural areas in low- and in middle-income countries [23].

## 6. Approaches to the Prevention of Unintentional Injuries

**Figure 1:**
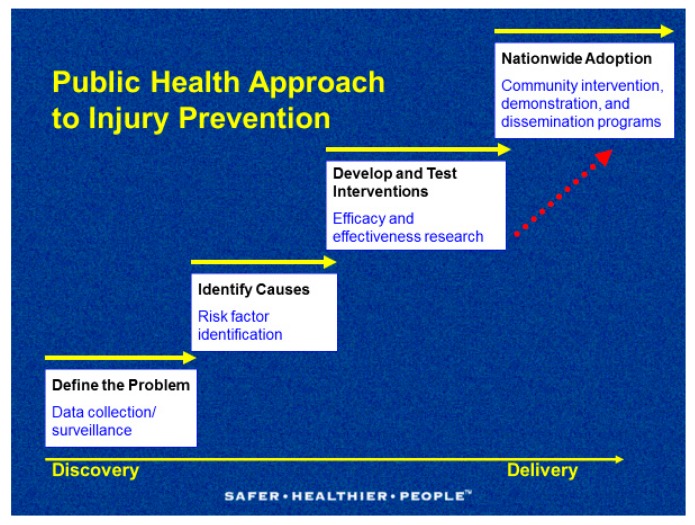
The public health approach to injury prevention. Source: National Center for Injury Prevention and Control, U.S. Centers for Disease Control and Prevention (CDC).

The traditional view of child and adolescent injuries as “accidents”, or random unpredictable events caused by fate, or God, has resulted in the historical neglect of designing and implementing prevention measures in public health. Today it is widely recognized that injuries are not accidents, they are predictable, preventable, and controllable. Worldwide, governments, public and private partners, Non Governmental Organizations (NGOs), and Foundations are increasingly aware of the strains that childhood injuries place on society. In response, they are strengthening data collection systems, identifying risk and protective factors, implementing and evaluating interventions, and disseminating evidence-based prevention strategies for nationwide adoption. These steps, which start with the discovery of facts and move to the delivery of programs, are part of a public health approach to injury prevention (Figure 1).

## 7. The Role of Public Health and Environment in Reducing Childhood Injury

Public health can be used to encourage individual behavior change on the part of children and caregivers, and environmental health can contribute to engineering solutions to make safe behaviors more likely and to improve the safety of products [30]. Evidence demonstrates that both public health and environmental science policies save lives, for example, regulations on poison packaging, restrictions on alcohol marketing and access, guard rails along highways, and policies that encourage seat belt and motorcycle helmet use. Addressing these problems comprehensively requires the participation of parents, teachers, law enforcement, governments, and NGOs. We also need to involve engineers, social workers, teachers, pediatricians, and child psychologists in efforts to uncover everyday solutions and apply “best practices” to reduce child injury.

It is clear from the articles in this compilation that unintentional injuries to children do not occur in isolation: They are determined by the choices children and their families make; the quality and design of their environment; the rules and regulations adopted and enforced by their society; the products they use; and by peer interactions and family dynamics at school and at home. Additionally, the quality of housing stock, vehicle safety standards, and the conditions of roads, as well as the social norms present in the community and the risks children take, all contribute to injuries.

Changes in injury patterns, including the likely causes of injury, and a child’s ability to respond to risks, are closely related to developmental stages over the life course. Exploring the physical environment is an important developmental task during infancy and early childhood. Children gradually encounter more and different injury risks at the same time they are developing the perceptual and cognitive abilities to adequately evaluate risky situations [31]. Risk-taking is an important part of the lives of children. We do not seek to eliminate all injury risks, but rather to manage and control them [32].

## 8. Advancing Child Injury Prevention

Several agencies around the world have developed national child injury prevention policies, strategies and/or plans of action that serve to guide efforts to prevent child injury and disability. For example WHO documents such as the *World Report on Road Traffic Injury Prevention, World Report on Violence and Health, World Report on Child Injury Prevention; INSPIRE: Seven Strategies for Ending Violence Against Children, Global Report on Drowning *, and the *USA’s National Action Plan for Child Injury Prevention, and the European Report on Child Injury Prevention* [1,21,27,33,34,35] all outline an agenda to prevent injuries and promote the safety of children and adolescents. For maximum impact, the WHO recommends that child injury policies, strategies, and action plans be concrete and contain clear objectives, priorities, timetables, and mechanisms for evaluation [1]. 

Among some of the common themes in national and international plans to advance child injury prevention are the following:

1. Integrate child injury prevention into a comprehensive approach to child health and development;
2. Develop, implement, and evaluate national child injury prevention policies and plans of action;
3. Implement evidence-based strategies to prevent and control injuries among children;
4. Evaluate existing programs, policies and strategies to determine what works best;
5. Improve efforts to raise public awareness of injuries to children and prevention strategies through traditional and modern communication channels;
6. Implement and disseminate child injury education and training programs in schools and in allied health professions;
7. Improve existing surveillance systems to address gaps in child injury data and improve access;
8. Define priorities for, and support research and evaluation on, the causes, effects, costs, and prevention of injury among children, including research to identify and reduce disparities;
9. Strengthen health care systems to support quality care of injured children in hospitals and clinics; and
10. Support the adoption and implementation of effective laws and policies that prevent child injuries.

Researchers, practitioners, and policy-makers are encouraged to take every available opportunity to implement one or more of these strategic themes to advance child injury prevention. It is hoped that the articles in this *IJERPH* compilation on child injury prevention will inspire public health and environment professionals around the world to look at the potential for improving child health through action on injury prevention. Based on the contents of these papers, it behooves us to consider applying these and other theories, methods, and strategies to prevent childhood injuries with as much scholarship and rigor as has been devoted to other non-communicable diseases. The time to act is now. What better future can we offer the children of the world than for them to grown up healthy without the threat of injury.
